# Enzyme Encapsulation in Liposomes: Recent Advancements in the Pharmaceutical and Food Sector

**DOI:** 10.3390/nano15151149

**Published:** 2025-07-24

**Authors:** Angela Merola, Lucia Baldino, Alessandra Procentese

**Affiliations:** Department of Industrial Engineering, University of Salerno, Via Giovanni Paolo II, 132, 84084 Fisciano, Italy; a.merola30@studenti.unisa.it (A.M.); aprocentese@unisa.it (A.P.)

**Keywords:** liposomes, enzymes encapsulation, drug administration, food preservation

## Abstract

Nanocarriers have found numerous applications in pharmaceutical and food sectors due to their unique physical and chemical properties. In particular, liposomes are the most extensively studied kind of nanoparticles for these applications. They are spherical colloidal systems characterized by lipid membranes enclosing an aqueous core. This versatile structure enables the incorporation of hydrophilic, hydrophobic, and amphiphilic molecules, making them optimal candidates for the controlled release of drugs and enzymes. Despite numerous promising applications, liposomes face challenges such as low colloidal stability, inefficient drug encapsulation, and high production costs for large-scale applications. For this reason, innovative methods, such as microfluidics, electroporation, and supercritical CO_2_, are currently being investigated to overcome these limitations. This review examines the recent applications of liposomes in enzyme encapsulation within the pharmaceutical and food sectors, emphasizing production challenges and emerging technological developments.

## 1. Introduction

Different types of nanocarriers are currently being investigated in the pharmaceutical and food sectors. The concept of nanoparticle-based therapies was conceived in 1955, and many different delivery systems have been demonstrated or developed since then. Although most applications are still limited to laboratory investigations, some have already entered the market or are currently in clinical trials [1]. Regarding nanomaterials in the food sector, they are mainly investigated to increase the food’s taste, extend the product’s shelf life, and accelerate ripening processes [2,3]. In both sectors, different kinds of nanostructures are currently being investigated, such as nanovesicles, solid–lipid nanoparticles, nanoemulsions, dendrimers, polymeric micelles, liposomes, hydrogels, and quantum dots [1,4]. All of these are characterized by advantages and disadvantages, which make them suitable for different applications.

**Figure 1:**
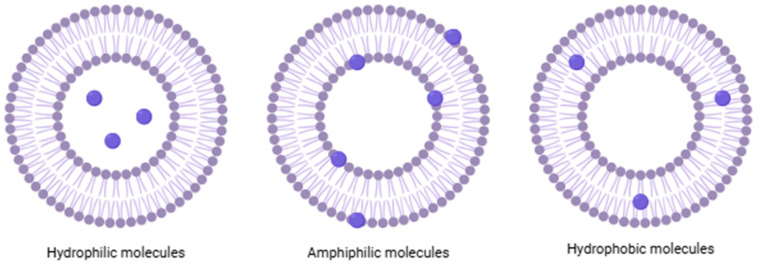
Schematic and simplified structure of liposomes loaded with different types of molecules.

Liposomes are colloidal spherical structures; their membrane can consist of one or more lipid bilayers, called lamellae, which enclose an aqueous core, with the polar heads oriented both inward and outward and both immersed in water [5]. What makes this structure unique is its ability to incorporate and release molecules with different solubility properties; hydrophilic molecules are located in the aqueous core, hydrophobic molecules are in the lipid bilayer, and amphiphilic molecules are located at the interface between water and the lipid layer (Figure 1) [6].

The amphiphilic nature of phospholipids in solution mimics natural cell membranes, facilitating the interaction between liposomes and mammalian cell membranes, which promotes efficient cellular uptake. Due to these properties, liposomes have been extensively studied as drug delivery systems. Their structural versatility, along with biocompatibility, biodegradability, non-toxicity, and non-immunogenicity, makes them excellent candidates for drug transport [6]. Despite their great success in numerous applications, liposomes also present some challenges, primarily related to poor colloidal stability in biological environments, caused by lipid hydrolysis and oxidation, particle fission and fusion, and the consequent loss of their active payload [7].

There are different liposome production methods that can be classified as conventional or innovative. Among the various traditional methods used to prepare liposomes, the most common ones include thin film hydration, reverse-phase evaporation, solvent injection, and detergent removal. These methods generally follow the following main steps: (i) dissolution of lipids in organic solvents, (ii) removal of the organic solvent, (iii) purification and isolation of liposomes, and (iv) analysis of the characteristics of final liposomes [8]. Among the innovative liposome preparation methods, the most currently investigated are microfluidic methods and supercritical CO_2_ methods [5].

Liposomes produced by traditional or innovative methods can be used to entrap nucleic acid, microorganisms, and antimicrobial agents [8]. Moreover, interesting results have been recently reported when enzymes are encapsulated in liposomes [8]. Indeed, thanks to the encapsulation in liposomes, the enzyme can be protected from different agents, and it can be delivered close to a specific substrate where the catalytic reaction can take place [5].

Several reviews are reported in the scientific literature regarding nanocarrier classification [7] and production method descriptions [5,6]. On the contrary, a data collection that highlights the advantages and disadvantages of encapsulation techniques with respect to the free molecule form is missing. This is mainly due to the different parameters (nanocarriers, production methods, encapsulated molecule) selected by different research groups, which make the data comparison challenging.

The present work focuses on enzymes encapsulated in liposomes using different techniques. After a brief description of the main and recent preparation methods, a comparison between the traditional approach (free enzyme form) and liposome use (encapsulated enzyme) is reported for both pharmaceutical and food sectors. In particular, thirteen recent articles published between 2020 and 2024 were analyzed for the pharmaceutical sector. Regarding the food sector, the number of articles published between 2020 and 2024 was limited; therefore, six articles published between 2015 and 2024 were analyzed.

### Main Preparation Methods

Among the various traditional methods used to prepare liposomes, the most common are thin film hydration and reverse-phase evaporation [6].

**Figure 2:**
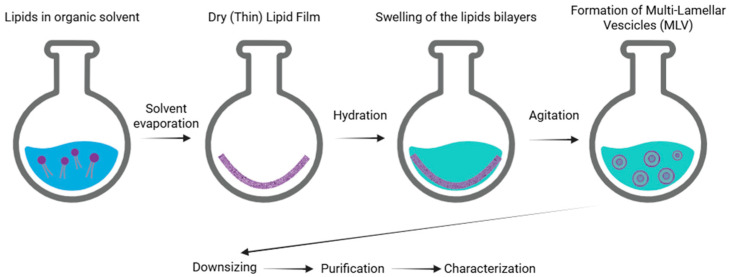
Schematic representation of the main stages of the thin film hydration method of liposomes.

Thin film hydration is the first production process described in the field of liposome technology (also known as the Bangham method). This is one of the simplest and most commonly used techniques [5,6]. The process is carried out as follows. Initially, phospholipids are dissolved in an organic solvent, such as dichloromethane, chloroform, ethanol, or a chloroform–methanol mixture, to ensure a homogeneous solution. The solvent is then evaporated, typically using a round-bottom flask, to form a thin lipid film. Once the solvent is completely removed, a dry and thin lipid film composed of stacked bilayers is obtained. Subsequently, an appropriate aqueous medium, typically distilled water or a buffer solution, is added, which facilitates the formation by causing swelling of the lipid bilayer stacks. The subsequent agitation/stirring of the sample promotes the formation of multilamellar vesicles. The final stages of the production process include liposome downsizing, purification, and characterization (Figure 2) [6]. The main drawbacks of the Bangham method include the complete removal of the organic solvent, the limited encapsulation efficiency, and difficulties in scaling-up the process for industrial production [5].

**Figure 3:**
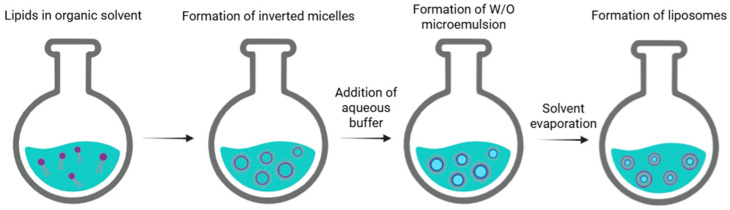
Schematic representation of the main stages of reverse-phase evaporation of liposomes.

Another conventional technique used for liposome preparation is reverse-phase evaporation. This method begins with a procedure similar to thin film hydration. Phospholipids are dissolved in an organic solvent, such as a mixture of diethyl ether/chloroform (1:1 *v/v*), diethyl ether/isopropyl ether, or chloroform/methanol (2:1 *v/v*), which facilitates the formation of inverted micelles. A defined amount of aqueous phase (such as a buffer) is then added to the solution. In this case, lipids arrange themselves at the interface between water and oil, creating a microemulsion in which water is dispersed in oil (W/O). This microemulsion can be further emulsified using mechanical methods or sonication to achieve a uniform dispersion. By employing rotary evaporation under vacuum, the organic solvent is gradually removed until a viscous gel is formed. Slow evaporation promotes the disruption of inverted micelles and facilitates the subsequent formation of liposomes. At a critical point, the gel collapses, and the excess of phospholipids in the solution rearranges around inverted micelles, forming a bilayer around the remaining water droplets, leading to the formation of liposomes (Figure 3) [5].

This method is advantageous for its high encapsulation efficiency, but it presents disadvantages such as exposing the compounds to sonication conditions and organic solvents, and it is also relatively time-consuming [6]. It has been employed to encapsulate both small and large molecules, such as RNA and various enzymes, while preserving their activity without causing degradation. However, a potential drawback of this method is the exposure of the encapsulated material to mechanical stress and organic solvents, which could lead to protein denaturation or DNA strand breakage [9].

In the realm of innovative systems, microfluidics is the most widely utilized technology. The microfluidic method for liposome production represents a significant innovation, as it enables precise control over both vesicle size and size distribution during their formation, without the need for additional post-production steps [10]. Fluid mixing occurs within a geometrically confined microenvironment characterized by a sub-micrometric length scale and a low Reynolds number. This technology allows for precise control over parameters affecting the assembly process, resulting in highly reproducible formulations. The laminar flow of aqueous and organic solvents, combined with predictable flow patterns in microfluidic mixing, ensures uniform particle size distributions. This technique also enables the adjustment of liposome size by setting appropriate operational conditions, such as total flow rate (TFR), flow rate ratio (FRR) between mixed solutions, total volume (TV) processed, and solvent choice [9]. In the microfluidic (MF) method, lipids dissolved in solvents such as ethanol or isopropanol are directed through microscopic channels with cross-sections ranging from 5 to 500 µm. The alcoholic phospholipid solution, confined between two aqueous flows within a microchannel, creates a laminar hydrodynamic flow and promotes diffusive mixing at the liquid interfaces, facilitating the self-assembly of lipids into vesicles. By precisely controlling the mixing and flow rates, this technique enables the production of small, monodisperse liposomal nanoformulations with tunable sizes and distributions, using low-toxicity solvents like ethanol. Furthermore, compared to conventional methods, MF-based processes require significantly less solvent, thus potentially reducing environmental impact and paving the way for a more sustainable approach [11]. Unlike traditional large-scale production methods, the final product does not require post-processing steps such as extrusion or sonication. Despite the considerable versatility and flexibility of the microfluidic method, its main limitations include the need for delicate mechanical handling and the complexity of scaling-up production [5,9]. Moreover, commercially available microfluidic devices are often extremely expensive and offer limited flexibility in terms of design and material selection, which significantly constrain their use in research applications [12].

## 2. Pharmaceutical Sector

### 2.1. Traditional Approach

Table 1 shows the state of the art related to different drug administrations. Currently, traditional approaches, such as intravenous or intranasal, are characterized by the following issues: immunogenicity, instability, and short half-time [11,13]. The use of liposomes able to address enzymes in specific sites could improve their stability and reduce the dose amount.

The mucopolysaccharidosis (MPS) disorders are caused by deficiencies of specific lysosomal enzymes involved in the catabolism of glycosaminoglycans. During mucopolysaccharides type I, glycosaminoglycans are accumulated. A possible solution to treat mucopolysaccharidosis type 1 (MPS I) is nasal administration of laronidase. Although nasal administration is a simple method, it is characterized by reduced residence time in nasal cavities; for this reason, several administrations are required [14].

Diseases such as rheumatoid arthritis or inflammation are mainly mediated by reactive oxygen species (ROS). SOD is an enzyme able to catalyze the dismutation of anion superoxide radical; thus, it is widely used in the treatment of these kinds of diseases. The intravenous and intraperitoneal administration of SOD, without a proper drug delivery system, has some drawbacks, such as a short half-life in the bloodstream, low accumulation in targeted areas, and rapid renal filtration [15,16].

In Acute Lymphoblastic Leukemia (ALL), cells do not have the L-asparaginase (ASNase) enzyme. Currently, ASNase of bacterial origin is administrated intravenously. However, it exhibits short therapeutic half-lives, plasma instability, and immunogenicity. Due to its bacterial origin, ASNase degradation by blood proteases is significant, and the generated epitopes are readily recognized by the immune system, promoting an immune response [17].

Liver metastases and colorectal cancer are characterized by the accumulation of NETs. When DNase I is intravenously administrated, a reduced accumulation of NETs has been reported [21]. However, intravenous administration of this viral vector can induce dose-dependent immunogenicity and genotoxicity, leading to acute systemic inflammation, coagulation defects, hepatic toxicity, and sensory neuron damage [13].

Cardiovascular disorders are characterized by blood clot formation. Currently, NK enzymes can dissolve blood clots administered intravenously. This method has advantages such as low toxicity, ease of production, and low cost. However, it also has significant limitations, including poor thrombus accessibility, denaturation in plasma, rapid clearance, hemorrhagic complications, and various side effects [11].

The last example concerns lung inflammatory diseases, where anion superoxide radicals are accumulated. When the SOD enzyme is administrated, the dismutation of anion superoxide radicals into molecular oxygen and hydrogen peroxide occurs. Currently, the enzyme is administrated without a proper transport system. This method has strengths such as higher activity, enhanced selectivity, and reduced side effects. Nevertheless, it also presents drawbacks, such as low physical and chemical stability, protein degradation, and immunogenicity [19].

### 2.2. Enzyme Encapsulation in Liposomes

The main issues described in the previous section could be minimized using a liposome system that addresses enzymes in specific sites, avoiding their recognition as exogenous, and thus their degradation in the blood system. Table 2 reports the state of the art related to enzyme encapsulation in liposomes for pharmaceutical applications.

Schuh et al. (2024) worked on laronidase encapsulation in liposomes for the MPS I therapy. The average size of liposomes loaded with laronidase was 103 nm, and they were characterized by a polydispersity index ≤ 0.2. Mucoadhesive liposomes were used to increase the bind to the nasal mucous membranes. In the mice with MPS I treated with nasal administration, a significant increase of enzymatic activity in the visceral organs and central nervous system was observed, confirming the effectiveness of liposomes transporting laronidase to the brain. A higher biodistribution in tissues such as lungs, heart, and cerebral cortex was achieved compared to the intravenous administration of the free enzyme during the same analysis period. Enzymatic activity in the spleen was found to be equivalent for both routes. Regarding in vivo tolerability, histological analysis of tissues collected from treated and untreated animals with the disease revealed no significant differences in any organs stained with hematoxylin–eosin. These findings demonstrated that the formulations were non-irritating and did not cause acute toxicity in the nasal mucosa, an important consideration for nasal administration [22].

SOD was encapsulated in liposomes by Costa and co-workers (2023). SOD is widely used to treat conditions associated with ROS, including rheumatoid arthritis, inflammatory processes, and ischemia-reperfusion injuries. The aim of this study was to develop a continuous, simple, single-step method to produce enzyme-loaded liposomes (SOD@Liposomes) using a glass-capillary microfluidic device. The primary objective was to achieve a scalable, reproducible, time- and cost-efficient process with high production yields. Furthermore, the goal was to ensure that the physicochemical properties of liposomes were comparable to those obtained by conventional methods, but with higher encapsulation efficiency and process yields. SOD@Liposomes prepared via the microfluidic method exhibited uniform size, with an average size of 135 ± 41 nm and a polydispersity index of 0.128 ± 0.010. Both SOD@Liposomes and empty liposomes showed ζ-potential values close to zero, as expected for PEG-coated liposomes. This neutral surface characteristic contributes to stability and reduces interaction with plasma proteins. The encapsulation efficiency of SOD@Liposomes was 59 ± 6%, significantly higher than that reported in previous studies. The enzymatic activity of SOD encapsulated in liposomes was maintained at 82 ± 3% of its initial activity. Furthermore, the effect of SOD-loaded liposomes and empty liposomes on cell viability was studied in two intestinal cell lines (Caco-2 and HT29-MTX). SOD@Liposomes demonstrated higher cell viability compared to empty liposomes in both cell lines, with 13 ± 9% and 14 ± 4% higher viability for Caco-2 and HT29-MTX, respectively. Moreover, SOD@Liposomes were deemed non-toxic, as cell viability remained above the safety threshold (>70%) even after prolonged exposures. Animal experiments conducted using the anthralin-induced ear edema model over 24 h evaluated the anti-inflammatory effect of SOD formulated in liposomes based on its antioxidant properties. The administration of SOD@Liposomes led to a 65 ± 8% inhibition of edema, whereas free SOD showed only a 20 ± 13% reduction. The superiority of SOD@Liposomes was attributed to the liposomes’ ability to prolong the enzyme’s half-life, protect it from rapid elimination, and the PEG coating that increases liposomal circulation in the bloodstream, enhancing bioavailability. Additionally, the enhanced permeability and retention effect allowed liposomes to accumulate in inflamed tissues, improving local distribution and therapeutic efficacy [23].

L-asparaginase (ASNase) is a key biological drug in the treatment of ALL. Encapsulation of ASNase in liposomes provides an effective solution to protect the enzyme from degradation by plasma proteases optimizing the drug pharmacokinetic profile. de Souza Guimarães and co-workers (2022) produced PEGylated liposomes for ASNase encapsulation, demonstrating their ability to deplete Asn and exhibiting in vitro activity against leukemic cells. The following two types of phospholipids were used to prepare the liposomal formulations: saturated 1,2-dimyristoyl-sn-glycero-3 phosphocholine (DMPC) and unsaturated 1,2-dioleoyl-sn-glycero-3-phosphocholine (DOPC). The best liposomal formulation was identified as DMPC/DSPE-PEG 10%, with a Z-average size ranging from 142 to 157 nm. Regarding polydispersity (PDI), the formulations presented values of PDI < 0.2, indicating monodispersity. Liposome permeability was assessed through the release of NH_3_, the product of ASNase activity. Low initial NH_3_ levels (up to 0.45 M) were attributed to the following factors: low ASNase encapsulation efficiency, low L-asparagine penetration into liposomes, and volatilization of the generated NH_3_. A significant increase in NH_3_ concentration after 120 min confirmed enzymatic activity and liposome permeability. Among the formulations, ASNase-DMPC/DSPE-PEG 10% exhibited the highest NH_3_ release, likely due to a packing mismatch between the lipid chains of DMPC and DSPE-PEG, resulting in a less compact and more permeable bilayer. The ASNase-DMPC/DSPE-PEG 10% formulation was tested against MOLT-4 leukemic cells and compared with free ASNase and ASNase-DMPC. No significant difference was observed between free ASNase and ASNase-DMPC, indicating that the enzyme retained its activity after encapsulation. However, ASNase-DMPC/DSPE-PEG 10% showed a lower IC50 value, highlighting the potential of this formulation to prolong enzyme circulation without triggering the immune system. The DMPC/DSPE-PEG 10% formulation stood out for its high efficiency in L-asparagine depletion and increased cytotoxicity against MOLT-4 leukemic cells compared to free ASNase and pure DMPC liposomes [24].

Penaloza et al. (2022) studied the potential of liposomal nanocarriers to improve the pharmacokinetic profile of DNase I. The following two approaches were evaluated to prolong the half-life of DNase-loaded liposomes: direct encapsulation of DNase I and immobilization of DNase I on the surface of liposomes. The hydrodynamic radius (RH) of DNase I, which is crucial for its encapsulation in drug delivery systems such as liposomes, was assessed. Only liposomes produced at a pH of 4 were found to be capable of acceptable protein encapsulation of 9.3 ± 1.9%. The protein was found to be stable at a pH of 4 in low ionic strength buffers (20 mM), showing an RH of approximately 9 nm in high ionic strength saline buffers (150 mM NaCl). Zeta potential indicated colloidal stability under these conditions. For the experiments, C57BL/6 mice were treated with labeled DNase I or HDNase I liposomes to evaluate pharmacokinetic parameters and biodistribution. Free DNase was rapidly cleared from plasma, whereas HDNase I liposomes exhibited a half-life of 7.1 h, compared to the native DNase half-life of 2.2 h, with a 32-fold increase in total exposure and detectability for up to 48 h. Biodistribution studies revealed that both formulations primarily accumulated in the liver and spleen, with a slight increase in accumulation for HDNase liposomes compared to empty liposomes [21].

Priya and co-workers (2024) encapsulated NK through the reverse phase evaporation technique. The targeting molecules used were RGD, a small tripeptide, and AM, a chimeric human monoclonal antibody. Drug release studies were performed for both targeted and non-targeted liposomal formulations. The targeted formulations (RGD-NK-LS and AM-NK-LS) exhibited lower release rates compared to the non-targeted formulation (NK-LS). The fibrinolytic potency of liposomal formulations was measured. The clot lysis percentage increased with the use of targeted liposomes, reaching 84.5% for AM-NK-LS after 12 h, compared to 64.4% for NK-LS. Platelet aggregation was observed with fibrinogen. Targeted liposomes (RGD-NK-LS and AM-NK-LS) showed lower absorbance compared to the control group, suggesting reduced platelet aggregation. Various in vivo tests were also conducted to evaluate the efficacy and safety of targeted nanodrugs, particularly liposomes. The bleeding time, utilized to identify hemorrhagic attributes of antithrombotic medications, and clotting time were analyzed in mice treated with different liposomes, highlighting differences in bleeding and clotting times compared to saline solution. Thrombolysis efficacy was assessed through doppler imaging and ultrasound; targeted liposomes (RGD-NK-LS and AM-NK-LS) showed a greater recovery of blood flow compared to non-targeted liposomes and saline solution. Additionally, targeting ability was studied using photoacoustic imaging (PAI), which revealed a higher accumulation of marked liposomes at the thrombus site when targeted, compared to non-targeted liposomes. Finally, histopathological analysis confirmed the absence of toxicity in the major vital organs of rats [25].

Costa and co-workers (2021) focused on SOD encapsulation followed by dry powder formulations (Lip-DPFs) using the supercritical fluid-assisted spray drying (SASD) technique. Stability tests were conducted on liposomal powders loaded with SOD and stored at 40 ± 5% relative humidity for 50 days, with the control of suspensions stored at 4 °C. After the storage period, liposomes showed a slight increase in size, but the encapsulation efficiency remained above 95%, demonstrating that trehalose prevented drug leakage and preserved enzymatic activity [26].

Ran and co-workers (2024) encapsulated SOD in multivesicular liposomes for the treatment of UC, a chronic bowel disease. SOD-loaded S-MVLs were prepared using a double emulsification process, achieving an encapsulation efficiency of 78.7 ± 2.6% and a particle size of 27.3 ± 5.4 μm. In vitro, the two-phase sustained-release profiles of SOD from S-MVLs were shown, which were the burst release phase within 4 h and the sustained-release phase. An amount of 20.1 ± 1.2% of SOD was released from S-MVLs within 4 h, while its release reached 75.2 ± 2.9% and 80.6 ± 1.9% at 96 h and 144 h, respectively. Liposomes were studied in murine models of dextran sulfate sodium salt (DSS) and trinitrobenzene sulfonic acid (TNBS)-induced colitis in C57BL/6 mice, demonstrating a significant therapeutic effect. In DSS-treated mice, weight loss was observed, along with an improved disease activity index and restored colon length and morphology compared to groups treated with empty MVLs or free SOD [27].

Sapkota and co-workers (2023) aimed to formulate papain-loaded liposomes and transferosomes, characterize the formulations, and investigate in vitro permeation using shed snake skin and Sprague Dawley rat skin as models for the stratum corneum and full-thickness skin, respectively. The thin film hydration method was used. Papain-loaded liposomes exhibited an encapsulation efficiency of 96.14 ± 0.29% and particle sizes of 150 ± 2 nm (pre-lyophilization), 476 ± 10 nm (post-lyophilization), and 161 ± 7 nm (post-lyophilization with trehalose). Moreover, the cytotoxicity was evaluated. After 72 h, at a concentration of 10 mg/mL of papain liposomes, a viability of approximately 27% was detected. At a higher concentration of 10 mg/mL, papain was able to permeate the stratum corneum barrier (shed snake skin model) [28].

Vanti and co-workers (2021) investigated the co-encapsulation of berberine chloride (BRB) and tariquidar (TAR) in nanoliposomes. This was carried out to enhance their solubility and optimize the desired effects of both the antineoplastic drug and the P-gp inhibitor. Transmission electron microscopy (TEM) analysis was performed on both the human leukemia cell line K562 and its doxorubicin (DOXO)-resistant counterpart, K562/DOXO, which was characterized by P-gp overexpression, to investigate the endocytosis properties of nanoliposomes loaded with HRP. The thin lipid film hydration technique was adopted. Nanoliposomes with a size of 161 ± 19 nm and a polydispersity index of 0.287 ± 0.051 were obtained. After 2 h of exposure to HRP-loaded nanoliposomes, most cells exhibited evident signs of necrosis, including cytoplasmic vacuolization, swollen mitochondria with cristae loss, chromatin disintegration, and multiple disruptions of the plasma membrane with cytoplasmic leakage. The internalization of HRP-loaded nanoliposomes was found to cause irreversible damage to the cytoplasmic membrane, severe mitochondrial dysfunction, and ultimately, cell death [29].

LOD encapsulated in liposomes by Wu and co-workers (2022) was designed to exploit its ability to catalyze lactate oxidation, generate H_2_O_2_, and create a hypoxic environment that promotes the activation of chemotherapy prodrugs, thereby enhancing the effectiveness of cancer therapy. The resulting liposomes exhibited an average hydrated particle size of approximately 140 nm and an encapsulation efficiency of 76%. Tumor cell oxidative stress was assessed and found to be increased, as evidenced by significant green fluorescence, thereby enhancing the efficacy of TPZ chemotherapy under hypoxic conditions. Furthermore, quantitative fluorescence analysis results confirmed that LOD/TPZ@Lips-LA effectively produced H_2_O_2_ and induced sufficiently strong tumor hypoxia, thereby activating tirapamycin and increasing oxidative stress in tumor cells. Treatment with LOD/TPZ@Lips-LA led to significant tumor suppression, achieving a tumor reduction rate of 81.1%, whereas LOD@Lips-LA and TPZ@Lips-LA groups exhibited lower reduction rates (42.1% and 58.1%, respectively). Moreover, mice treated with LOD/TPZ@Lips-LA demonstrated prolonged survival compared to the other groups, whereas in the control groups, tumors rapidly progressed, leading to death within 30 days [30].

Sommonte and co-workers (2022) encapsulated lysozyme in liposomes to develop improved drug delivery systems. The used encapsulation method was the microfluidic system. Liposomes were produced with a particle size of the first trial with the optimal ratio of 181 ± 12 nm and a polydispersity index of 0.17 ± 0.04. The achieved encapsulation efficiency was 40.89 ± 6.19%. The release study was conducted under dynamic dialysis for three days at 37 °C in phosphate-buffered saline (pH 7.4). The obtained release profile showed that L-LPs 0.5 mg mL^−1^ formulation enabled the release of 93.36 ± 5.85% of the total payload within 72 h of incubation. The profile exhibited an initial rapid release during the first 4 h of incubation, reaching 44.82 ± 5.16%, followed by a zero-order kinetics release (R^2^ = 0.9985), ensuring a controlled and sustained release over time [12].

To address intracellular mycobacterial infections, Bartlett and co-workers (2024) developed a cocktail of four enzymes that catalytically attacked three layers of the mycobacterial envelope. This cocktail was delivered to macrophages through a targeted liposome. EC1 was designed using the following four enzymes: LysA, LysB, isoamylase, and α-amylase. LysA hydrolyzes peptidoglycan, LysB cleaves the arabinogalactan-mycolic acid linkage, while α-amylase and isoamylase degrade capsular polysaccharides by targeting α-1,4 and α-1,6 glycosidic bonds, respectively. Liposome diameter and polydispersity index were analyzed, but they were not reported. The efficacy of EC1 was then directly compared with common frontline antibiotic therapies on a set of pathogenic strains. The minimum inhibitory concentrations (MICs) of EC1 in combination with four standard-of-care (SoC) antibiotics were measured against the same NTM and *M. tuberculosis* strains to assess whether their interactions were synergistic, additive, indifferent, or antagonistic in inhibiting mycobacterial growth. EC1 exhibited a strong synergistic effect with standard-of-care antibiotics against *Mycobacterium tuberculosis* and nontuberculous mycobacteria (NTM), significantly reducing the required concentrations of amikacin, cefoxitin, and rifampicin. FICI analysis confirmed the absence of antagonism and demonstrated synergy for nearly all tested strains, with only *M. tuberculosis* H37Rv showing additive effects [31].

Kim and co-workers (2022) developed pH-sensitive twin liposomes (TLs) containing quercetin (QER, as a model prodrug) and laccase (LAC, as a bioactive catalyst for generating toxic QER through catalytic oxidation) in separate compartments. QER and LAC were encapsulated using the liposome magnetoporation method. The following four types of SLs were prepared: QER@GDEAP−biotin−SLs, LAC@GDEAP−avidin−SLs, QER@GDOCA−biotin−SLs, and LAC@GDOCA−avidin−SLs. QER/LAC@GDEAP−TLs were then obtained through an avidin−biotin reaction between QER@GDEAP−biotin−SLs and LAC@GDEAP−avidin−SLs, achieving a production yield of 82 ± 6.0 wt.%. Similarly, QER/LAC@GDOCA−TLs were prepared, resulting in a production yield of 79 ± 9.0 wt.%. QER/LAC@GDEAP−TLs exhibited high efficacy in generating ROS at a pH of 6.8, but not at a pH of 7.4. In contrast, QER/LAC@GDOCA−TLs, free QER, free LAC, and the mixture containing QER@GDEAP−biotin−SLs and LAC@GDEAP−avidin−SLs were less effective in generating ROS at both a pH of 7.4 and a pH of 6.8. QER/LAC@GDEAP−TLs demonstrated excellent antitumor efficacy against MDA-MB-231 cancer cells in an acidic environment. Tumor volume was measured in MDA-MB-231 tumor-bearing nude mice following intravenous injection of QER/LAC@GDEAP−TLs, revealing a significant tumor volume reduction. Ten days post-injection, the tumor volumes in mice treated with QER/LAC@GDEAP−TLs were approximately 3.8, 2.5, 3.9, 4.9, 4.6, and 4.9 times lower compared to mice treated with QER/LAC@GDOCA−TLs, the mixture containing QER@GDEAP−biotin−SLs and LAC@GDEAP−avidin−SLs, free QER/LAC mixture, free QER, free LAC, and saline, respectively. QER/LAC@GDEAP−TLs demonstrated superior efficacy in in vivo tumor inhibition, suggesting that LAC-mediated rapid enzymatic activation of QER at the tumor site may represent a promising strategy for targeted cancer therapy [32].

## 3. Food Sector

### 3.1. Traditional Approach

The use of enzymes in the food sector dates to wine, bread, and beer production [33]. Currently, enzyme properties related to food process improvement, tasting enhancement, and shelf life rising are investigated [3]. However, all these processes, without a proper delivery system, are still characterized by issues summarized in Table 3.

Cheese maturation is a slow, costly, and not fully controllable phase in the production process of ripened cheeses [3]. To address this issue, enzymes are added. The addition of enzymes can induce early proteolysis by hydrolyzing caseins into soluble peptides. The premature breakdown of caseins disrupts the ordered structures of the enzymes, resulting in soft curds that are unusable in subsequent acidification stages [34].

Ricin is a type II ribosome-inactivating protein that inhibits ribosomes, and it is considered a potential food contaminant. Currently, innovative fluorescent methods have been developed that use aptamers as recognition elements for ricin detection. However, as noted by Xiao and co-workers (2016), these methods still have certain limitations, including the complexity of the fluorescent dye modification process and issues related to the use of organic fluorophores, such as photodegradation, poor photostability, and sensitivity to environmental changes [35].

Protease has been used by Ashie and co-workers (2002) for the development of meat tenderization techniques. The enzymatic degradation method enhances meat tenderness by breaking down muscle fibers through the use of exogenous enzymes. However, this process can lead to the formation of undesirable flavors and textures due to substrate specificity and the breakdown of major muscle proteins [36].

*Escherichia coli* O157 demonstrates a remarkable ability to adhere, colonize, and form biofilms on various food surfaces, posing a significant challenge to food hygiene practices. PK is known to promote the dispersion of bacterial biofilms, facilitating the release of cells and small aggregates. However, this process may enable bacteria to colonize new areas and restart the biofilm development cycle [37].

The enzyme-linked immunosorbent assay (ELISA) is a reliable technique for detecting specific biomarkers, leveraging the targeted interaction between antigens and antibodies and the signal generated by a color change. It can be used for the food quality control procedure [38].

ADH is an intracellular enzyme capable of catalyzing the dehydrogenation of both endogenous and exogenous alcohols. It is used to catalyze the conversion of alcohol present in food into acetaldehyde, in order to reduce the negative effects of excessive ethanol intake. Currently, ADH is primarily derived from animal liver, with issues of poor stability, high cost, and low catalytic efficiency, thus significantly limiting its application in the food industry [39].

### 3.2. Enzyme Encapsulation in Liposomes

The main disadvantages related to the use of enzymes in the food industry could be avoided using liposomes. In the present section, a collection of the main scientific papers regarding enzyme encapsulation in liposomes for food application is reported; a summary is presented in Table 4.

The purpose of the study conducted by Jahadi and co-workers (2017) was to investigate the potential of liposomes used as carriers for the encapsulation of LEF in order to accelerate the maturation of Iranian salted white cheese. The encapsulation efficiency of LEF liposomes was 26.5%, with an average diameter of the loaded liposomes of 189 nm. Among the aspects analyzed, the total solids content in the cheeses was measured, and a statistically significant decrease in total solids was observed after the enzyme addition. This phenomenon was caused by the binding of water to the surface of liposomes and the absorption of water by the curd matrix, leading to increased moisture in the cheeses and a reduction in total solids. No significant differences were found in salt levels between the cheese samples with free enzymes (FF), encapsulated enzymes (LEF), and the control cheese during the different maturation days (1, 10, 20, and 30 days). This result highlights that the addition of enzymes, both encapsulated and free, did not affect the salt content or distribution gradients during maturation, confirming that enzyme use preserves salt levels without requiring modifications to the salting process. The cheeses with LEF exhibited the most significant increase in WSN/TN (water-soluble nitrogen/cheese total nitrogen, expressed as a percentage) and TCA/TN (non-protein nitrogen/total nitrogen, expressed as a percentage of TN) ratios compared to the other samples during maturation, indicating that the use of liposomes in cheeses with LEF intensified proteolysis to a greater extent than in cheeses with free enzymes or the control. Based on the WSN/TN and TCA/TN values, the addition of LEF could reduce the maturation time by 10 and 20 days, respectively [34].

Ricin is a type II protein consisting of two chains (RTA and RTB) linked by disulfide bonds. It is a potential food contaminant that can be detected through GOD. Men and co-workers (2018) employed liposomes able to carry out large amounts of GOD to amplify ricin signal detection. Liposomes were produced with an average size of approximately 165 ± 16 nm, whereas Zeta potential was −27.4 ± 1.5 mV. Different experimental conditions were investigated to achieve optimal performance in ricin toxin (RTB) detection. The number of liposomes was crucial, with the maximum response achieved at a concentration of 2.6 mg/mL. The best reaction times for the hybrid probe/RTB interaction and GOD/glucose interaction were 50 and 40 min, respectively. The proposed method demonstrated high sensitivity, with a good linear relationship between the fluorescence change (ΔF) and RTB concentration in the range of 0.25–50 μg/mL. The detection limit was 190 ng/mL, a competitive result compared to traditional methods. The system selectivity was excellent, clearly distinguishing RTB from other proteins and interfering substances such as vitamins and metal ions. To verify the practicality of the method, ricin toxin content in castor seeds was analyzed, comparing the results with an ELISA kit. The values obtained (3.38 ± 1.11%) were consistent with those from the kit (2.94 ± 0.06%) and the literature [2].

Kim and co-workers (2017) aimed to develop methods for softening beef to create products that elderly individuals can easily chew and swallow. After protease encapsulation, the particle size increased to 365 ± 76 nm, which was significantly larger than that of empty liposomes. Zeta potential of the liposome-coated protease was −13 ± 1.9 mV, with no significant differences compared to the uncoated protease. The color properties of the beef showed a progressive darkening with increasing reaction time, with the uncoated protease exhibiting a darker shade than the liposome-coated protease. Generally, color variations were more pronounced in the control and with uncoated protease, while liposome-coated protease showed less sensitivity to time-induced color changes. The water holding capacity (WHC) of the beef decreased with increasing reaction time. The control had a WHC of 79.39 ± 2.77% at 12 h, which dropped to 75.71 ± 0.97% by 48 h, while that of the liposome-encapsulated protease decreased from 79.16 ± 2.17% to 75.86 ± 1.93%. No significant differences were observed between the control and the liposome-encapsulated protease. Both unencapsulated and liposome-encapsulated proteases showed no significant differences from the control during the first 12 h, but after 24 h, the WHC of both protease types significantly decreased as reaction time increased [40].

*Escherichia coli* O157:H7 is an enteric pathogen that causes severe gastrointestinal diseases and forms biofilms on food surfaces, compromising hygiene. An innovative method to inhibit pathogenic biofilms is the smart release of thyme essential oil (TO) and PK from liposomes. Cui et al. (2016) designed liposomes co-loaded with PK and TO. The optimal liposomes had an average size of 170 ± 4 nm and a negative Zeta potential close to −30 mV, preventing aggregation and precipitation. The temporal elimination curves of various formulations against *E. coli* O157:H7 biofilms were evaluated in vitro. It was found that the combination of unencapsulated PK and TO was more effective in eliminating biofilms compared to the individual treatments throughout the observation period of 5 days. The practical efficacy of PK/TO liposomes was tested on cucumbers stored at three different temperatures (5 °C, 15 °C, and 25 °C) for 3 days. At 5 °C, no significant reduction of biofilms was observed, while at 15 °C, antibacterial activity was more pronounced, with reductions of 1.61 log (300 mg/mL) and 2.32 log (400 mg/mL). Finally, at 25 °C, despite an increase in bacterial population in control samples, liposomes gradually reduced the biofilms, showing greater efficacy compared to the lower temperatures. The best results were obtained at 15 °C, likely due to the optimal release of PK and TO from liposomes. Regarding food quality evaluation, this was assessed after 1 and 3 days of storage at 15 °C. After 1 day, no significant changes were observed in either color parameters or sensory quality compared to fresh cucumbers. However, after 3 days, the treated cucumbers showed a significant decrease in brightness and an increase in yellow value compared to the control samples. The visual appearance and odor were partially altered, with a residual TO scent perceived negatively. Nevertheless, the overall sensory quality scores remained above the acceptability threshold [41].

As already reported, ELISA is a robust strategy for detecting specific biomarkers through antigen–antibody interactions, with the signal generated by a color change. However, its sensitivity is limited since only a small amount of enzyme catalyzing a chromogenic substrate can be modified on the immunocomplex. To overcome this limitation, one solution involves combining the immunocomplex with multiple enzymes. In this context, enzyme-encapsulated liposomes are among the best candidates for signal amplification, thereby improving sensitivity. The principle of the proposed aptasensor, based on enzyme-loaded liposomes, involves the following three stages: immobilization of the capture probe, hybridization between the aptamer, capture probe, and detection probe, and the combination of double-stranded DNA with enzyme-loaded liposomes. The ssDNA-1, modified with an amino group at its 5′ end, was conjugated to carboxyl-modified magnetic beads. The OTA aptamer then hybridized with ssDNA-1 and ssDNA-2, forming dsDNA. Thanks to the biotin at the 3′ end of ssDNA-2, HRP-loaded liposomes were linked via a biotin–avidin–biotin bond, forming the MBs–dsDNA–liposome handle-like probe. In the presence of OTA, the aptamer binds to it, forming a G-quadruplex, releasing ssDNA-2 and the liposomes, which were magnetically separated. The liposome lysis released HRP, catalyzing the oxidation of TMB by H_2_O_2_, resulting in a color change from colorless to blue, visible to the naked eye. TEM confirmed the formation of well-structured liposomes (diameter ~100 nm). The feasibility of the probe was verified, as a high signal was observed only when all components were present, demonstrating the sensor’s specificity and the validity of its principle. The sensor exhibited high specificity, effectively distinguishing OTA from interfering substances (AFB1, OTB, OTC), with a visible color change occurring only in the presence of OTA due to the specific interaction between the aptamer and OTA. When applied to maize samples, the sensor detected OTA concentrations consistent with reference values. Standard recovery rates (98.5–106.1%) and relative standard deviations (5.3–6.9%) confirmed the reliability of the method [41].

ADH was encapsulated in liposomes using the film evaporation dynamic high-pressure microfluidization method, as reported by Zhang and co-workers (2024). As an intracellular enzyme responsible for catalyzing the dehydrogenation of both endogenous and exogenous alcohols, ADH is utilized to convert alcohol in food into acetaldehyde, thereby mitigating the adverse effects of excessive ethanol intake. EGCG is a potent compound with hepatoprotective properties, capable of reducing alcohol-induced liver damage. The EGCG-ADH complex can effectively enhance the biological activity of ADH due to the interaction between EGCG and ADH. Since the Zeta potential of the samples was negative, ranging from −2.5 to −5.0 mV, electrostatic forces played a role in their interactions. The median diameter (D50 value) of blank liposomes and ADH liposomes ranged from 20 to 500 nm, while that of EGCG-ADH liposomes ranged from 200 to 1000 nm. In contrast, D50 values of liposomes encapsulating EGCG alone ranged from 3000 to 10,000 nm, exceeding the nanoscale range. The release efficiency of LC-EGCG-ADH liposomes and the EGCG-ADH complex was evaluated during simulated gastrointestinal digestion. The release of LC-EGCG-ADH was higher than that of free EGCG-ADH over two hours of digestion in simulated gastric and intestinal fluids. EGCG-ADH was found to be unstable in acidic conditions, undergoing rapid degradation, whereas it remained stable in neutral and basic environments. Consequently, free EGCG-ADH degraded in the simulated gastric fluid, exhibiting the lowest enzymatic activity. Liposomal encapsulation allowed EGCG-ADH to be entrapped within the liposome network, reducing direct contact with gastric juice and preserving its structural stability. The results demonstrated that liposomes effectively resisted gastric acidity and modulated the release of EGCG-ADH. In simulated intestinal fluid, liposome-encapsulated EGCG-ADH exhibited significantly higher enzymatic activity than its free form. Furthermore, liposomes delayed the hydrolysis of EGCG-ADH in the gastrointestinal tract [43].

## 4. Conclusions

The encapsulation of enzymes in liposomes represents a cutting-edge technology with potential applications across various sectors, including the pharmaceutical and food industries. Liposomes can enhance the efficacy, stability, and biodistribution of enzymes. In particular, in the pharmaceutical field, liposomes enable innovative solutions for enzyme delivery, improving therapeutic effectiveness in challenging conditions such as genetic diseases and chronic inflammations.

In the food sector, encapsulated enzymes contribute to accelerate industrial processes, such as cheese ripening, while enhancing food quality and safety.

Despite the advancements, challenges remain in scaling up this technology to industrial levels. Nevertheless, with ongoing technological progress in the field, liposomes represent a versatile and evolving platform with the potential to revolutionize numerous application areas.

## Figures and Tables

**Table 1:** Advantages and disadvantages of the current drug administration methods.

Molecule	Drug Administration Method	Advantages	Disadvantages
Laronidase (a recombinant version of α-L-iduronidase) [14]	Nasal administration	Enzymes deliver to limited-access tissues due to direct communication with the central nervous system through the olfactory pathway	Short residence time in the nasal cavity.
Superoxide dismutase (SOD) [15,16]	Intravenous administration and intraperitoneal administration	Higher activity and selectivity and lower side effects	Short half-life in the blood stream (~6 min in rats and 25 min in humans), low accumulation in affected areas, and rapid renal filtration.
L-asparaginase (ASNase) [17]	Intravenous administration	Asn enables protein synthesis in leukemic cells	Short therapeutic half-lives, plasma instability and immunogenicity. Due to its bacterial origin, the degradation of ASNase by blood proteases is considerable, and the generated epitopes are easily recognized by the immune system, promoting an immune response.
Deoxyribonuclease I (DNase I) [13,18]	Intravenous administration	DNase I has been positively correlated with a reduced accumulation of neutrophil extracellular traps (NETs) in tumors and serum	Intravenous administration of this viral vector can induce dose-dependent immunogenicity and genotoxicity, leading to acute systemic inflammation, coagulation defects, hepatic toxicity, and sensory neuron damage. Short serum half-life.
Nattokinase (NK) [11]	Intravenous administration	Low toxicity, ease of production, and low cost	Limited drug accessibility to the thrombus, drug denaturation in plasma, rapid drug clearance, hemorrhagic complications, and a range of side effects.
Superoxide dismutase (SOD) [19]	Pulmonary administration	Higher activity and selectivity and lower side effects	Low physical and chemical stability, protein degradation, and immunogenicity.
Superoxide dismutase (SOD) [20]	Intraperitoneal administration	Targeted effects with reduced toxicity	Low drug loading capacity and rapid release profiles.

**Table 2:** Enzyme encapsulation in liposomes for pharmaceutical applications.

Enzyme	Liposomes Production Method	Applications	Liposomes Characterization	Main Results
Laronidase [22]	Lipid thin film method followed by microfluidization	Illness: MPS I Attached cell or substrate: Degradation of glycosaminoglycans	ζ-potential +30 mV Polydispersity index 0.101 Size 103 ± 3 nm Mucoadhesive force: sample 6 ± 0.1 mN, control 4 ± 0.1 mN	In vitro: MPS I fibroblasts treated with the formulation presented a significant increase in enzyme activity when compared to the control, reaching up to 50% of wild-type mice values (~5000 nmol/h/mg protein). In vivo: Nasal administration showed a significant increase in enzyme activity. In the lungs, enzyme activity increased from 3 nmol/h/mg protein to 20 nmol/h/mg protein.
Superoxide dismutase (SOD) [23]	Nanoprecipitation method, using a modified co-flow microfluidic glass-capillary device	Illness: Ear edema Attached cell or substrate: SOD catalyzes the dismutation of anion superoxide radical in molecular oxygen and hydrogen peroxide	ζ-potential −0.6 ± 0.2 mV Polydispersity index 0.128 ± 0.01 Size 135 ± 41 nm Encapsulation efficiency 59 ± 6% Enzymatic activity 82 ± 3%	In vitro: Cytotoxicity was assessed. SOD@Liposomes showed significantly higher cell viability compared to empty liposomes, with viability at 13 ± 9% and 14 ± 4% for two intestinal cell lines. In vivo: SOD@Liposomes presented a higher edema inhibition (65 ± 8%) compared to SOD in its free form (20 ± 13%).
L-asparaginase (ASNase) [24]	Lipid thin film method followed by electroporation	Illness: ALL. Attached cell or substrate: ASNase catalyzes the hydrolysis of L-asparagine (Asn) in the bloodstream resulting from the products aspartic acid (Asp) and ammonia (NH_3_).	The best liposome formulation was DMPC/DSPE-PEG 10% ζ-potential −2.5 mV Polydispersity index 0.190 ± 0.020 Size 142 ± 10 nm	In vitro: ASNase-loaded DMPC/DSPE-PEG 10% systems enhanced cytotoxicity against the MOLT-4 leukemic cell line compared to free ASNase. IC_50 values for pure ASNase (0.000376 ± 0.000027 U/mL) were less favorable compared to those of ASNase-DMPC/DSPE-PEG 10% (0.000267 ± 0.000029 U/mL).
Deoxyribonuclease I (DNase I) [21]	Microfluidic micromixing	Illness: Atherosclerosis-related thrombosis, cerebral ischemia, and neurovascular dysfunction Attached cell or substrate: NETs	Values at pH 4: ζ-potential ~1 mV Encapsulation efficiency 9.3 ± 1.9% The enzyme was immobilized on the liposome. Two initial protein concentrations (250 and 500 μg/mL) were tested at two molar ratios of C18-PEG4-NHS/HDNase I (1:8 and 1:16). A reduction in initial hydrophobic modification of DNase (HDNase I) concentration led to increased liposomal size (133 ± 5 and 105 ± 10 for HDNase at 250 and 500 μg/mL, respectively) and had no effect on the polydispersity index (~0.34) of liposomes	In vitro: The plasma concentration of free DNase was observed to rapidly decay after 15 min and was undetectable after 6 h. Pharmacokinetic parameters showed a terminal elimination half-life of 7.1 h for HDNase, which was notably higher than that for native DNase I (2.2 h).
Nattokinase (NK) [25]	Reverse phase evaporation	Illness: Cardiovascular disorders Attached cell or substrate: NK dissolves blood clots	Arginyl-glycyl-aspartic acid (RGD) and abciximab (AM) were used as a target ζ-potential of: NK-LS −06.65 ± 0.58 mV RGD-NK-LS −08.96 ± 0.96 mV AM-NK-LS −09.24 ± 1.59 mV Polydispersity index of: NK-LS 0.178 ± 0.010 RGD-NK-LS 0.261 ± 0.085 AM-NK-LS 0.275 ± 0.054 Size of: NK-LS 163 ± 4 nm RGD-NK-LS 173 ± 6 nm AM-NK-LS 178 ± 5 nm Encapsulation efficiency of: NK-LS 73.20 ± 1.76% RGD-NK-LS 69.96 ± 2.46% AM-NK-LS 69.76 ± 1.42%	In vitro Drug Release Study: NK-LS 83.96 ± 2.05%, RGD-NK-LS 80.3 ± 2.94%, AM-NK-LS 77.96 ± 1.24% after 72 h. Clot Lysis Assay: NK 42.93 ± 4.20%, NK-LS 46.26 ± 5.45%, RGD-NK-LS 64.40 ± 3.05%, AM-NK-LS 84.50 ± 2.60%. In vivo Bleeding Time Analysis: NK 143.33 ± 16.99 s, NK-LS 108.33 ± 19.39 s, RGD-NK-LS 130.33 ± 11.89 s, AM-NK-LS 133.66 ± 9.46 s. In vivo Clotting Time Analysis: NK 141.66 ± 10.27 s, NK-LS 132.66 ± 12.11 s, RGD-NK-LS 128.33 ± 6.23 s, AM-NK-LS 129.66 ± 12.97 s.
Superoxide dismutase (SOD) [26]	Lipid thin film method followed by extrusion method and then dried using supercritical CO_2_-assisted spray-drying (SASD)	Illness: Lung inflammatory diseases Attached cell or substrate: SOD catalyzes the dismutation of anion superoxide radicals in molecular oxygen and hydrogen peroxide	Polydispersity index 0.211 ± 0.009 Size 117 ± 3 nm Encapsulation efficiency > 95%	In vitro: SOD_Lip-DPFs exhibited a mass median aerodynamic diameter (MMAD) of 1.6 ± 0.4 µm. A low MMAD indicates that the majority of the particles were smaller in size. The fine particle fraction was 98.6 ± 0.4%, indicating the percentage of particles that reached the lower respiratory tract (aerodynamic size < 5 µm), specifically the terminal bronchi.
Superoxide dismutase (SOD) [27]	Double emulsification process	Illness: Ulcerative colitis (UC) Attached cell or substrate: SOD plays a crucial role in mitigating gut mucosal injury against oxidative stress	Size 27.3 ± 5.4 μm Encapsulation efficiency 78.7 ± 2.6%	In vitro: SOD released from multivesicular liposomes (S-MVLs) exhibited the following biphasic release profile: an initial rapid release (20.1% within 4 h) followed by a sustained release phase, reaching 75.2% at 96 h and 80.6% at 144 h. In vivo: S-MVLs have proven effective in alleviating ulcerative colitis in DSS-treated mice by reducing oxidative stress through the scavenging of reactive oxygen species (ROS). They improved the disease activity index and restored colon length and morphology compared to treatments with empty MVLs or free SOD.
Papain [28]	Lipid thin film method	Illness: Treatment of hypertrophic scars and keloids	ζ-potential −50.09 ± 3.66 Size: pre-lyo 150 ± 2 nm, post-lyo 475 ± 10 nm, post-lyo with trehalose 161 ± 7 nm Encapsulation efficiency 96.14 ± 0.29%	In vitro: After 72 h, at a concentration of 10 mg/mL of papain liposomes, a viability of approximately 27% was detected. At a higher concentration of 10 mg/mL, papain was able to permeate the stratum corneum barrier (shed snake skin model).
Horseradish peroxidase (HRP) [29]	Lipid thin film method	Illness: The multidrug resistance (MDR)	Size 161 ± 19 nm Polydispersity index 0.287 ± 0.051	In vitro: The two cell lines were incubated with HRP-loaded nanoliposomes and subsequently analyzed by transmission electron microscopy analysis to assess their behavior after 15 and 120 min of exposure, comparing it with the controls. HRP-loaded nanoliposomes enter K562 and K562/DOXO cells via receptor-mediated endocytosis, causing irreversible membrane damage and mitochondrial dysfunction, ultimately leading to necrosis and cell death after 2 h.
Lactate oxidase (LOD) [30]	Lipid thin film method	Illness: Cancer Attached cell or substrate: The natural enzyme, lactate oxidase, catalyzes the oxidation of lactate by O_2_ to produce H_2_O_2_, a ROS	Size around 140 nm Encapsulation efficiency 76%	In vitro: To optimize the feed ratio between LOD/TPZ@ lips and bacteria, the effect of LOD/TPZ@lips on bacterial viability was measured. The bacterial Cell Counting Kit-8 (CCK-8) results indicated that the excessive LOD/TPZ@lips had a significant impact on the growth of lactobacillus (LA). In vivo: Mice treated with LOD/TPZ@Lips-LA demonstrated prolonged survival compared to the other groups; whereas, in the control groups, tumors rapidly progressed, leading to death within 30 d.
Lysozyme [12]	Microfluidic system	Objective: Developing improved drug delivery systems	Polydispersity index (first trial with the optimal ratio) 0.17 ± 0.04 Size (first trial with the optimal ratio) 181 ± 11 nm Encapsulation efficiency 40.89 ± 6.19%	In vitro: The obtained release profile showed that L-LPs 0.5 mg/mL formulation allowed for 93.36 ± 5.85% release of the total payload until 72 h of incubation.
Endolytix Cocktail 1 (EC1) was designed using four enzymes: LysA, LysB, isoamylase, and α-amylase [31]	Microfluidic system	Illness: Nontuberculous mycobacteria infection Attached cell or substrate: LysA hydrolyzes peptidoglycan, LysB cleaves the arabinogalactan-mycolic acid linkage, while α-amylase and isoamylase degrade capsular polysaccharides by targeting α-1,4 and α-1,6 glycosidic bonds, respectively	-	In vitro: EC1 exhibited a strong synergistic effect with standard-of-care antibiotics against Mycobacterium tuberculosis and nontuberculous mycobacteria (NTM), significantly reducing the required concentrations of amikacin, cefoxitin, and rifampicin.
Laccase [32]	Magnetoporation method	Illness: Tumor	Four types of single liposomes (SLs) were prepared: QER@GDEAP−biotin−SLs, LAC@GDEAP−avidin−SLs, QER@GDOCA−biotin−SLs, and LAC@GDOCA−avidin−SLs. QER/LAC@GDEAP−TLs were obtained through an avidin−biotin reaction between QER@GDEAP−biotin−SLs and LAC@GDEAP−avidin−SLs, achieving a production yield of 82 ± 6.0 wt.%. Similarly, the QER/LAC@GDOCA−TLs were prepared, resulting in a production yield of 79 ± 9.0 wt.%	In vitro: QER/LAC@GDEAP−TLs were highly effective in generating ROS at a pH of 6.8, but not at a pH of 7.4. In contrast, QER/LAC@GDOCA−TLs, free QER, free LAC, and the mixture containing QER@GDEAP−biotin−SLs and LAC@GDEAP−biotin−SLs were less effective in generating ROS at both a pH of 7.4 and a pH of 6.8. In vivo: QER/LAC@GDEAP−TLs demonstrated superior efficacy in in vivo tumor inhibition, suggesting that LAC-mediated rapid enzymatic activation of QER at the tumor site may represent a promising strategy for targeted cancer therapy.

**Table 3:** Main problems and current approaches related to food spoilage.

Enzyme	Application	Current Approach	Disadvantages
Flavourzyme (LEF) [34]	Cheese ripening	Enzymes are added to accelerate cheese ripening	The addition of enzymes can cause early proteolysis, hydrolyzing caseins into soluble peptides
Ricin [35]	Food contamination	New fluorescent methods have been developed that utilize aptamers as recognition elements for the detection of ricin	Issues associated with organic fluorophores, including photobleaching, poor photostability, and sensitivity to the external environment
Protease [36]	Development of meat tenderization techniques	The enzymatic degradation technique enhances meat tenderness	Enzymatic degradation may result in undesirable flavor and texture
Proteinase K (PK) [37]	*E. coli* O157 exhibits a high ability to adhere, colonize, and form biofilms on a variety of food surfaces	PK is known to promote the dispersion of bacterial biofilms	The release of cells and small aggregates could allow bacteria to colonize new areas, restarting the biofilm development cycle
Horseradish peroxidase (HRP) [38]	Identification of some specific contaminants	The enzyme-linked immunosorbent assay (ELISA) is a robust strategy for detecting specific biomarkers	The sensitivity is limited because only a small amount of enzyme catalyzing the chromogenic substrate can be incorporated into the immunocomplex
Alcohol dehydrogenase (ADH) [39]	ADH is used to catalyze the conversion of alcohol present in food into acetaldehyde in order to reduce the negative effects of excessive ethanol intake	ADH produced primarily from animal liver is still used	Issues of poor stability, high cost, and low catalytic efficiency, thus significantly limiting its application in the food industry

**Table 4:** Enzyme encapsulation in liposomes for food applications.

Enzyme	Liposome Production Technique	Applications	Liposome Results	Food Application Results
Flavourzyme (LEF) [3]	Heating method	The acceleration of Iranian white brined cheese ripening	Size 189 nm Encapsulation efficiency 26.5%	Water-soluble nitrogen/cheese total nitrogen (WSN/TN) was evaluated. After 30 days, values of approximately 35% were observed for the encapsulated enzyme, ~25% for the non-encapsulated enzyme, and ~23% for the control.
Glucose oxidase (GOD) [2]	Lipid thin film method followed by sonication	GOD is used to detect the B-chain of ricin, a potential food contaminant	ζ-potential −27.4 ± 1.5 mV Size 165 ± 16 nm Encapsulation efficiency 59%	The proposed method demonstrated high sensitivity, with a good linear relationship between the fluorescence change (ΔF) and RTB concentration in the range of 0.25–50 μg/mL.
Protease [40]	Two-stage homogenization procedure	Techniques have been developed to enhance meat tenderness	ζ-potential −13 ± 1.9 mV Polydispersity index 0.41 ± 0.07 Size 365 ± 76 nm	The non-coated protease (NCP) exhibited a significantly higher tyrosine content (0.21 ± 0.03 ppm) at 24 h, but the tyrosine levels in both NCP and coated protease (CP) were the same after 24 h.
Proteinase K (PK) [41]	Lipid thin film method	*E. coli* O157:H7 shows a high ability to attach, colonize, and form biofilms on a variety of food surfaces, which causes a serious problem for food hygiene	The values of PK/Thyme Oil liposomes (1:20) were: Size 175 ± 4 nm Polydispersity index 0.301 ± 0.009 ζ-potential −33.5 ± 2.8 mV Entrapment efficiency of thyme oil 34.3 ± 3.1%	The treatment with PK/TO liposomes exhibited a desirable bactericidal activity during 3 days of incubation.
Horseradish peroxidase (HRP) [42]	Ethanol injection method	The developed colorimetric aptasensor was applied to detect ochratoxin A (OTA) concentration in corn samples. Sensitive liposome-based colorimetric aptasensor was developed, a dumbbell-shaped probe was designed, including magnetic beads, double-stranded DNA, and enzyme-encapsulated liposome	Size 100 nm Liposomes were well distributed and there were no ruptured liposomes	Each liposome contained a large amount of HRP. Thus, when the liposome was lysed by adding the mixed solution of 3,3′,5,5′-tetramethylbenzidine (TMB) and H_2_O_2_, a large amount of HRP was released. HRP can catalyze the H_2_O_2_-mediated oxidation of TMB and, hence, can result in the color change of the system from colorless to blue. Consequently, the concentration of OTA can be observed by naked eyes easily.
Alcohol dehydrogenase (ADH) [43]	Film evaporation-dynamic high-pressure microfluidization	ADH is employed to catalyze the conversion of alcohol in food into acetaldehyde to reduce the negative effects of excessive ethanol intake	Epigallocatechin gallate (EGCG) was co-encapsulated with ADH in liposomes. ζ-potential ranging from 2.5 to 5.0 mV Size 200–1000 nm	The results demonstrated that liposomes effectively resisted gastric acidity and modulated the release of EGCG-ADH. In simulated intestinal fluid, liposome-encapsulated EGCG-ADH exhibited significantly higher enzymatic activity than its free form.
